# Neuroimaging analysis reveals distinct cerebral perfusion responses to fasting‐postprandial metabolic switching in Alzheimer's disease patients

**DOI:** 10.1111/cns.70014

**Published:** 2024-09-11

**Authors:** Runzhi Li, Zhizheng Zhuo, Zeshan Yao, Zhaohui Li, Yanli Wang, Jiwei Jiang, Linlin Wang, Wenyi Li, Yanling Zhang, Jun Sun, Junjie Li, Yunyun Duan, Yi Liu, Hongyuan Shao, Yang li, Yechuan Zhang, Jinglong Chen, Hanping Shi, Hui Huang, Yaou Liu, Jun Xu

**Affiliations:** ^1^ Department of Neurology, Beijing Tiantan Hospital Capital Medical University Beijing China; ^2^ Department of Neurology, Shanxi Provincial People's Hospital The Fifth Clinical Medical College of Shanxi Medical University Taiyuan China; ^3^ Shanxi Key Laboratory of Brain Disease Control, Shanxi Provincial People's Hospital Taiyuan China; ^4^ Department of Radiology, Beijing Tiantan Hospital Capital Medical University Beijing China; ^5^ Jingjinji national center of technology innovation Beijing China; ^6^ BioMind Inc Beijing China; ^7^ Department of Neurology, First Hospital Shanxi Medical University Taiyuan China; ^8^ Department of Engineering Science, Institute of Biomedical Engineering University of Oxford Oxford UK; ^9^ Department of Geriatric Medicine, Guangzhou First People's Hospital, School of Medicine South China University of Technology Guangzhou China; ^10^ Department of Gastrointestinal Surgery, Beijing Shijitan Hospital Capital Medical University Beijing China; ^11^ Department of Clinical Nutrition, Beijing Shijitan Hospital Capital Medical University Beijing China; ^12^ Key Laboratory of Cancer FSMP for State Market Regulation Beijing China; ^13^ Department of Head and Neck Surgical Oncology, National Cancer Centre/National Clinical Research Centre for Cancer/Cancer Hospital, Chinese Academy of Medical Sciences and Peking Union Medical College Beijing China

**Keywords:** Alzheimer's disease, cerebral blood flow, cognitive function, fasting, intermittent fasting

## Abstract

**Aims:**

Extended fasting–postprandial switch intermitting time has been shown to affect Alzheimer's disease (AD). Few studies have investigated the cerebral perfusion response to fasting–postprandial metabolic switching (FMS) in AD patients. We aimed to evaluate the cerebral perfusion response to FMS in AD patients.

**Methods:**

In total, 30 AD patients, 32 mild cognitive impairment (MCI) patients, and 30 healthy control individuals (HCs) were included in the quantification of cerebral perfusion via cerebral blood flow (CBF). The cerebral perfusion response to FMS was defined as the difference (ΔCBF) between fasting and postprandial CBF.

**Results:**

Patients with AD had a regional negative ΔCBF in the anterior temporal lobe, part of the occipital lobe and the parietal lobe under FMS stimulation, whereas HCs had no significant ΔCBF. The AD patients had lower ΔCBF values in the right anterior temporal lobe than the MCI patients and HCs. ΔCBF in the anterior temporal lobe was negatively correlated with cognitive severity and cognitive reserve factors in AD patients.

**Conclusions:**

AD patients exhibited a poor ability to maintain cerebral perfusion homeostasis under FMS stimulation. The anterior temporal lobe is a distinct area that responds to FMS in AD patients and negatively correlates with cognitive function.

## Graphical abstract

How could the cerebral perfusion response to fasting‐postprandial metabolic switching in Alzheimer's Disease (AD)? Difference cerebral blood flow (ΔCBF) of arterial spin labeling imaging between fasting and postprandial states was used to reflect cerebral perfusion response to fasting‐postprandial metabolic switching. AD participants had poor ability to maintain cerebral perfusion homeostasis; AD had lower ΔCBF values in the right anterior temporal lobe than mild cognitive impairment patients and healthy controls; ΔCBF in the anterior temporal lobe showed a negatively correlated with cognitive function in AD. ΔCBF in the anterior temporal lobe can be a potential radiological marker to reflect the cognitive function in fasting‐postprandial metabolic switching in AD.

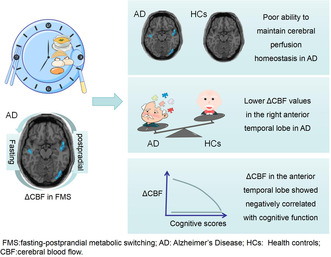

## 1 INTRODUCTION

Alzheimer's disease (AD) is recognized as a brain energy disorder caused by Aβ deposition and tau protein hyperphosphorylation.^1^ An approximately 18% brain energy deficit was reported in the early AD phase with disruption of glycolysis and oxidative phosphorylation.^1^ Intermittent fasting, which extends the fasting–postprandial switch interval, is an effective therapy for promoting energy metabolism in AD animal models.^1^, ^2^ Intermittent fasting decreased neuropathology and ameliorated cognitive deficits in AD mouse models.^3^, ^4^ The fasting–postprandial metabolic switch (FMS) is the basic cycle of a metabolic challenge that induces ketosis or a low glucose concentration (fasting) followed by a recovery period (postprandial). An increase in the fasting–postprandial switch intermitting time may optimize brain function and resilience throughout the lifespan, with a focus on the neuronal circuits involved in cognition and mood.^2^, ^5^

FMS impacts multiple signaling pathways that promote neuroplasticity and resistance of the brain to injury and disease.^2^ A better understanding of the adaptive neuronal mechanism of FMS may help to better understand intermittent fasting as a therapeutic method in AD. FMS induces a switch in the neuronal metabolic state by switching the major cellular fuel source from ketone‐dominant or low glucose metabolism to a glucose‐dominant metabolic pattern.^6^, ^7^ FMS induces adaptive cellular stress‐response signaling pathways in neurons involving neurotrophic factors, autophagy, DNA repair proteins, protein chaperones, and mitochondrial biogenesis.^2^, ^8^ The above molecular biological mechanisms of FMS in AD have been partially elucidated at the cellular level.

However, the pattern of neuroreactivity to FMS in AD and MCI patients has still not been addressed. Neuroimaging technology has been used to better understand neural reactivity and alterations in metabolic processes.^9^ Neurovascular coupling, a process that allows rapid neuronal activity in response to increased cerebral blood flow (CBF),^10^, ^11^ constitutes the basis for the “functional mapping” of CBF in the brain. Therefore, CBF is a sensitive way to reflect neural activity via cerebral perfusion. Arterial spin labeling (ASL) is a noninvasive MRI technique for measuring CBF by magnetically labeling water molecules as endogenous tracers.^12^, ^13^ ASL‐evaluated CBF, which reflects the adaptive neuronal response to FMS in individuals with AD and MCI, could help to reveal dynamic energy metabolism disorders in AD patients.

To address the above questions, we conducted an exploratory prospective experiment evaluating cerebral perfusion patterns to FMS in AD patients, MCI patients, and healthy control individuals (HCs). To gain initial insight into the possible relationships among the cerebral response to FMS, cognitive function, and vascular‐related factors, we assessed the associations between perfusion changes due to FMS and cognitive scores, blood pressure, body mass index (BMI), insulin resistance, fasting glucose, and C‐peptide.

## 2 METHODS

### 2.1 Study registrations and patients

The participants included in this study were from a subgroup of the Chinese Imaging, Biomarkers and Lifestyle (CIBL) Study of Alzheimer's Disease and were recruited between September 2020 and September 2021.^14^, ^15^ The participants in the FMS experiments were required to be aged ≥40 years. Participants with existing neurological conditions, including significant psychiatric disorders or central neurological diseases, were excluded. Participants who consumed drugs or substances that may affect CBF on the day of MRI acquisition and those whose images demonstrated excessive head movement and scanner artifacts were excluded. The diagnostic criteria for HCs were (1) normal activities of daily living, (2) a normal range of cognition reflected by objective cognitive examination, and (3) no history of alcoholism or neurological diseases, such as primary central nervous system tumors, severe traumatic brain injury, or severe mental system diseases. Patients with AD and MCI were grouped according to the clinical criteria for possible and probable AD^16^ and MCI.^17^

This study collected clinical variables, including education, occupation, BMI, and cognitive assessment, including the minimum mental state examination (MMSE), Montreal Cognitive Assessment (MoCA), and cognitive reserve factors. The cognitive reserve factors related to both education and occupation were calculated using categorical principal component analysis,^18^ in which a high score indicates high cognitive reserve. Peripheral blood pressure was measured after 10 min of rest.

Peripheral blood was collected from participants in a fasting state. Insulin and C‐peptide concentrations were determined using a Roche automated immunoassay analyzer (cobas 8000c801) and the sandwich principle electrochemiluminescence method. Glucose concentrations were measured using the glucohexokinase method and a Roche biochemical analyzer (Cobas c702). Insulin resistance was indexed by the homeostatic model assessment for insulin resistance (HOMA‐IR).^19^

This study was performed in accordance with the Declaration of Helsinki and approved by the Institutional Review Board of Beijing Tiantan Hospital, Capital Medical University. All participants provided informed written consent before participating in the study. The clinical trial registration number was ChiCTR2100051526.

### 2.2 Experimental design

**FIGURE 1:**
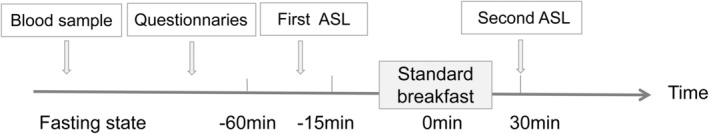
Time sequence of the experimental protocol. ASL: Arterial spin labeling.

FMS was designed as 1 cycle of at least 10 h of overnight fasting followed by a standard breakfast. All participants fasted for at least 10 h on the day of the experiment, and drugs or substances that may affect cerebral perfusion were avoided. The participants subsequently underwent the first ASL imaging scan and structural MRI scan in the fasting state, yielding the fasting‐state CBF. After the MRI scan, the standard breakfast was 500 kcal, with a carbohydrate, fat, and protein intake ratio of 5:3:2,^20^, ^21^, ^22^ which was finished within 15 minutes (Figure 1). Water was provided ad libitum. All the participants finished breakfast within 15 min (Figure 1). A second ASL imaging 30 min after the standard breakfast procedure yielded the postprandial‐state CBF. The CBF difference (ΔCBF) between the fasting and postprandial states was used to reflect the cerebral perfusion response to FMS.

The second ASL scan was designed with 30 min intervals after eating. Based on the evidence, 30 min after breakfast may be the most obvious time for brain metabolic switching in fasting–postprandial switching. Postprandial glucose and insulin levels most significantly increase 30 min after various combinations of meals.^23^ In addition, eating reduces brachial blood pressure and central blood pressure between 30 min and 45 min.^24^, ^25^ The experiment was designed with an interval of 10 h of overnight fasting on the day of the experiment. Two important findings indicate that a postprandial duration of more than 14 h is associated with metabolic‐related damage, such as increases in weight, blood pressure, and atherogenic lipids.^26^, ^27^ Therefore, a fasting interval of more than 10 h was considered relatively healthy and used as the time point in the present study.

### 2.3 MR image acquisition and processing

3D T1‐weighted (T1W) imaging and ASL imaging were performed for each subject on a 3 T MR scanner (Siemens Premier; GE Healthcare) using a 48‐channel head coil. During each scan, the participants rested quietly in the supine position without movement. They were asked to keep their eyes closed without specific thoughts and were pointed forward. The 3D T1W scans were acquired using the following parameters: repetition time (TR)/echo time (TE) TE = 7.3/3.0 ms; flip angle (FA) = 12 deg; field of view (FOV) = 256*256 mm^2^; acquisition matrix = 256 × 256; slice thickness = 1.0 mm; slice number = 176; and scan time = 4 min 56 s. The ASL scans were acquired using a 3D pseudocontinuous ASL sequence and the following parameters: axial acquisition, TR/TE = 4849/10.6 ms; FOV = 220*220 mm^2^; 8 arms and 512 points per arm; slice thickness = 4 mm; slice number = 36; and postlabel delay = 2025 ms. These are the default settings for GE ASL imaging.

CBF images were produced using the default postprocessing pipeline embedded in the GE‐MR console (AW Server, GE). The equation for quantifying the CBF in ml/100 g/min is shown in the Data S1. Image processing was performed using the SPM12 toolbox (http://www.fil.ion.ucl.ac.uk/spm) with the following steps: (1) coregistration of the M0 image (obtained from the raw ASL image) with the T1W image; (2) normalization of T1W images to the Montreal Neurological Institute (MNI) template and segmentation of the T1W image into gray matter (GM), white matter, and cerebral spinal fluid probability images; (3) generation of the GM mask by thresholding the GM probability image at 0.7; (4) warping of the CBF image into the MNI space using the forward transformation matrix derived from coregistration and normalization parameters; and (5) smoothing of the CBF image with a Gaussian kernel of full width at half maximum at 6 mm and resampling of the CBF image into an isotropic 3 × 3 × 3 mm^3^ voxel. At the voxel level, voxel‐based analysis was performed to identify the altered CBF regions induced by FMS. ΔCBF, computed by subtracting the fasting CBF from the postprandial CBF, was computed with an in‐house MATLAB script by applying the GM mask.

### 2.4 Statistical analysis

Statistical analyses were performed using the SPSS software (IBM SPSS Statistics for Windows, version 25) and the DPABI toolbox (version 6.1, http://rfmri.org/DPABI), and the results were visualized using the BrainNet Viewer toolbox (version 1.7, https://www.nitrc.org/projects/bnv/). For measurement data (Table 1), parametric analysis of variance (ANOVA) or nonparametric Kruskal–Wallis H test was applied, followed by post hoc two‐sample tests. For categorical data, the chi‐square test was used. *p* < 0.05 from two‐sided tests was considered to indicate statistical significance.

**TABLE 1:** Baseline characteristics of the participants.

Group	ALL	AD	MCI	HCs	*p* value*
*N*	92	30	32	30	
Age, years	62.5 ± 6.4	63.9 ± 6.0	62.8 ± 6.6	61.2 ± 6.3	0.266
Sex, Male, *n* (%)	65 (70.7%)	22 (71.0%)	24 (72.7%)	19 (63.3%)	0.697
BMI, kg/m^2^	24.7 ± 3.18	24.7 ± 3.82	24.6 ± 3.18	24.9 ± 2.3	0.949
Education, years, median (IQR)	11.0 (9.0–12.0)	9.0 (8.5–12.0)	12.0 (9.0–12.0)	10.0 (9.0–13.0)	0.579
Occupation^a^, *n* (%)					0.291
Physical labor	34 (37.4%)	14 (41.2%)	8 (23.5%)	12 (35.3%)	
Physical and mental labor	35 (38.5%)	8 (27.6%)	14 (43.8%)	13 (43.3%)	
Mental labor	22 (24.2%)	7 (31.8%)	10 (45.5%)	5 (22.7%)	
Vascular related factor					
Mean blood pressure	96.0 ± 12.7	96.6 ± 14.3	94.7 ± 12.8	96.9 ± 11.2	0.778
Systolic blood pressure	128.9 ± 17.3	131.1 ± 20.4	127.4 ± 16.6	128.3 ± 14.8	0.703
Diastolic blood pressure	79.6 ± 11.4	79.3 ± 12.2	78.4 ± 11.7	81.2 ± 10.5	0.634
Insulin, mIU/mL	9.7 ± 7.1	10.0 ± 6.6	10.4 ± 9.0	8.9 ± 5.2	0.710
HOMA‐IR	2.8 ± 4.8	2.2 ± 1.4	3.8 ± 7.9	2.4 ± 1.8	0.414
Glucose, mmol/L	5.7 ± 2.0	5.4 ± 1.1	6.0 ± 2.9	5.6 ± 1.4	0.496
C‐peptide, nmol/L	0.8 ± 0.3	0.9 ± 0.4	0.7 ± 0.3	0.8 ± 0.3	0.373
Hypertension, *n* (%)	40 (43.5%)	12 (40.0%)	16 (50.0%)	12 (40.0%)	0.654
Diabetes, *n* (%)	12 (13.0%)	3 (10.0%)	4 (12.5%)	5 (16.7%)	0.741
Hypercholesterolemia, *n* (%)	35 (38.0%)	9 (30.0%)	13 (40.6%)	13 (43.3%)	0.530
Smoking, *n* (%)	13 (14.1%)	4 (13.3%)	2 (6.2%)	7 (23.3%)	0.154
History of stroke	10 (10.9%)	4 (13.3%)	3 (9.4%)	3 (10.0%)	0.867
Cognitive function					
MMSE, median (IQR)	26.0 (20.0–29.0)	18.0 (11.2–22.0)	27.0 (24.0–28.2)	29.0 (27.0–29.0)	<0.001 ^ab^
MoCA, median (IQR)	21.0 (14.0–24.0)	11.5 (7.2–15.0)	21.0 (18.0–23.2)	25.0 (23.0–28.0)	<0.001 ^abc^
Cognitive Reserve factor	0.00 ± 1.00	0.03 ± 1.05	−0.09 ± 0.82	0.07 ± 1.14	0.818
Gray matter CBF, ml/100 g/min					
Fasting state	42.4 ± 8.4	38.5 ± 9.2	43.6 ± 6.6	45.1 ± 8.0	0.005 ^ab^
Postprandial state	41.6 ± 8.9	37.5 ± 9.8	43.1 ± 7.9	44.1 ± 7.8	0.007 ^ab^
Gray matter ΔCBF, ml/100 g/min	−0.8 ± 4.0	−1.1 ± 4.2	−0.5 ± 3.3	−1.0 ± 4.6	0.806

Abbreviations: AD, Alzheimer's disease; BMI, body mass index; CBF, cerebral blood flow. HOMA‐IR: Homeostatic Model Assessment for Insulin Resistance; HCs, healthy controls; IQR, interquartile range; MCI, mild cognitive impairment; MMSE, minimum mental state examination; MoCA, Montreal Cognitive Assessment.

^a^ One patient had missing values.

*Significant difference after post hoc comparison: a: AD patients vs. MCI patients; b: AD patients vs. HCs; c: HCs vs. MCI patients. ΔCBF: postprandial CBF‐fasting CBF.

The analysis of the perfusion data involved three steps. First, to investigate perfusion pattern responses to FMS, we used voxelwise paired t tests to compare fasting CBF and postprandial CBF among AD patients, MCI patients, and HCs. False discovery rate (FDR) correction (*q* < 0.05) with a GM mask and a cluster size >30 voxels were considered to indicate statistical significance. Statistically significant cerebral areas were defined as the ΔCBF areas responsive to FMS. Second, to investigate the differences in the ΔCBF among the three groups (AD patients, MCI patients, and HCs), we used voxelwise ANOVA and post hoc two‐sample tests. Finally, to explore the associations between ΔCBF and MMSE, MoCA, cognitive reserve factors, blood pressure, BMI, insulin resistance, fasting glucose, and C‐peptide, linear regression analyses were performed with age and sex as covariates. A T value >2.3, *p* < 0.01, and cluster size >30 voxels were considered to indicate statistical significance.^28^ Additionally, subgroup analyses of linear regression in participants with or without hypertension were performed to explore the effect of hypertension on ΔCBF associations. All the ΔCBF areas were reported according to the Anatomical Automatic Labeling (AAL) atlas^29^ using the “Anatomical ROIs Analysis” toolbox of DPABI.

## 3 RESULTS

### 3.1 Baseline characteristics of the study participants

Ninety‐seven participants were initially enrolled in the study. Five participants were excluded because of excessive head movement (*n* = 3) or scanner artifacts (*n* = 2) in the images. In total, 92 participants were enrolled in the study, among whom 70.7% were male (Table 1). The mean age of all participants in the three groups was 62.5 (±6.4) years. Based on the consensus diagnosis, the participants were divided into three groups: AD (*n* = 30), MCI (*n* = 32) and HC (*n* = 30). There were no significant differences in age, sex, education, occupation or vascular‐related factors, including BMI, fasting glucose, insulin, HOMA‐IR, C‐peptide, hypertension, diabetes, hypercholesterolemia, smoking, or the incidence of stroke, among the three groups. AD patients presented lower MMSE and MoCA scores than MCI patients and HCs (all *p* < 0.001). MCI patients presented lower MoCA scores than HCs (21.0 vs. 25.0, *p* < 0.001). The numbers of subjects in the AD group receiving pharmacological interventions were as follows: cholinesterase inhibitors (*n* = 19), N‐methyl‐D‐aspartate (NMDA) receptor antagonists (*n* = 9), and other agents (sodium oligomannate, nicergoline) (*n* = 4). In total, 20 AD patients (74.1%) received pharmacological interventions, and 3 patients were missing information on pharmacological interventions. The numbers of subjects in the MCI group receiving pharmacological interventions were as follows: cholinesterase inhibitors (*n* = 9), NMDA receptor antagonists (*n* = 2), and other agents (sodium oligomannate, nicergoline) (*n* = 0). In total, 7 MCI patients (23.3%) received pharmacological interventions, and 2 patients did not provide information on pharmacological interventions. The brain gray matter CBF in both the fasting and postprandial states in the AD patients (38.5 and 37.5 mL/100 g/min) was lower than that in the MCI patients (43.6 and 43.1 mL/100 g/min) and HCs (45.1 and 44.1 mL/100 g/min). The whole‐brain gray matter ΔCBF was −1.1 mL/100 g/min in the AD group, −0.5 mL/100 g/min in the MCI group, and −1.0 mL/100 g/min in the HC group, with no significant differences among the groups (Table 1).

### 3.2 Perfusion pattern responses to metabolic switching during fasting and postprandial states

**FIGURE 2:**
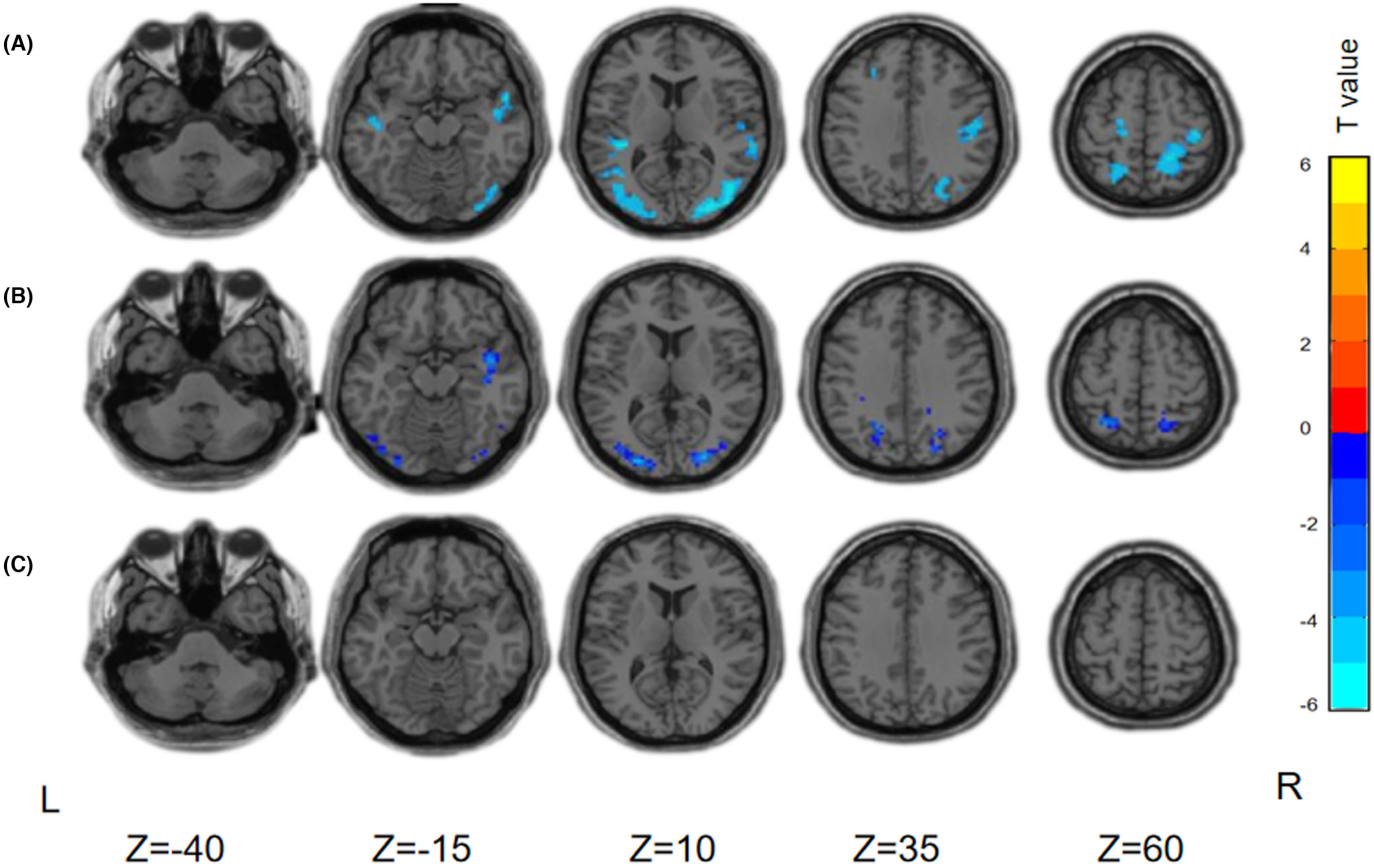
Significant ΔCBF areas corresponding to FMS in AD patients, MCI patients and HCs (paired *t*‐test, FDR corrected, *p* < 0.05, cluster size >30 voxels). (A) AD group, (B) MCI group, and (C) HC group. FDR, false discovery rate; L, left; R, right. Z: Horizontal brain sections corresponding to Montreal Neurological Institute coordinates.

Based on the voxelwise paired *t*‐test, a negative ΔCBF was found in the anterior temporal lobe, part of the occipital lobe, and the parietal lobe of patients in the AD group (Figure 2A). MCI patients had an intermediate ΔCBF change pattern between AD patients and HCs. In the MCI group, negative ΔCBF was found in brain areas similar to those in the AD group. However, the brain regions were smaller in size, and the magnitude of negative ΔCBF was lower (Figure 2B). No significant changes in the ΔCBF area were found in the HC group (Figure 2C). Table S1 shows the details of the location of the significant ΔCBF areas in the AD and MCI groups.

### 3.3 Between‐group differences in ΔCBF

**FIGURE 3:**
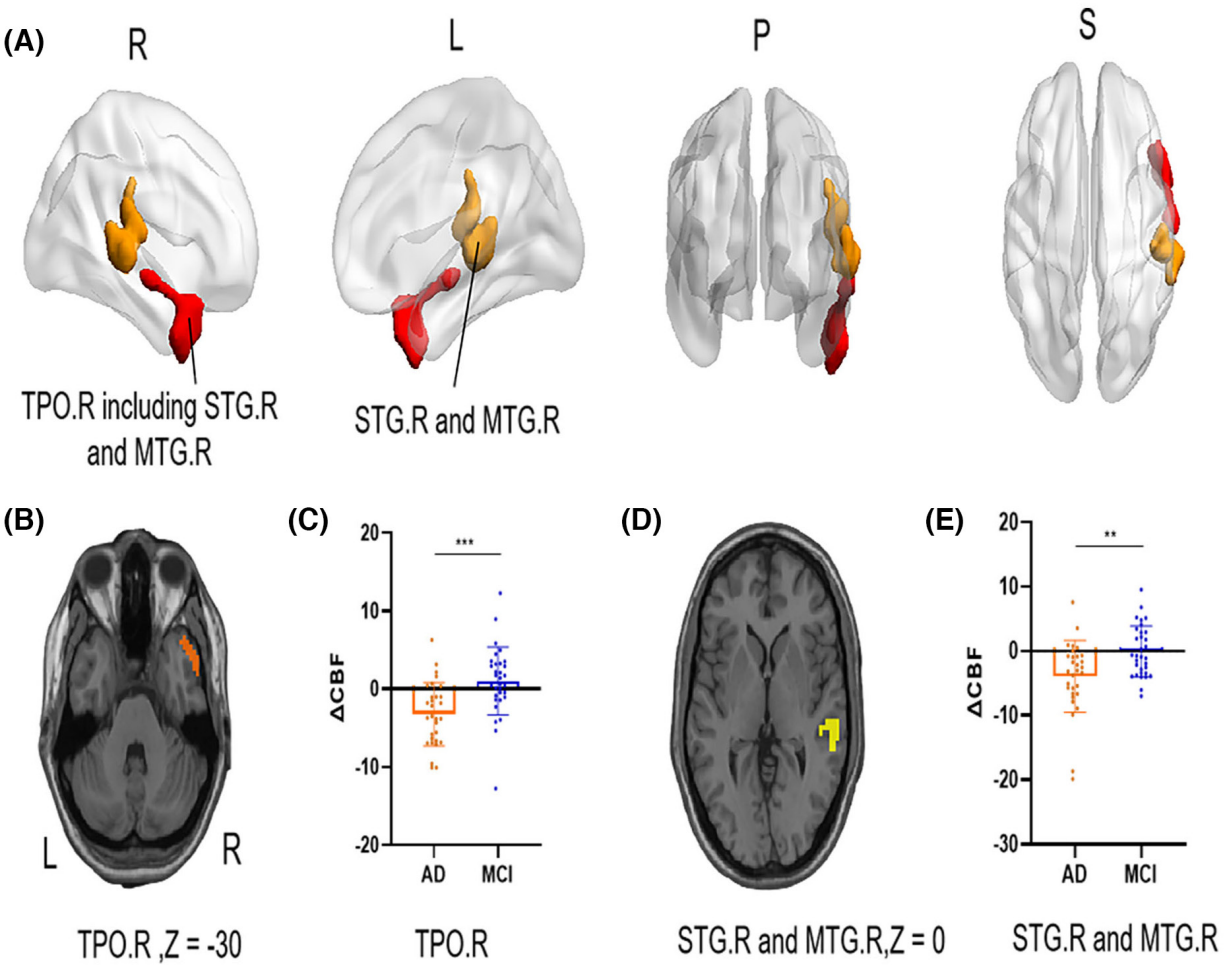
Significantly different ΔCBF areas between the AD and MCI groups. (A) Surface‐based brain map of different ΔCBF areas between AD patients and MCI patients. (B) Anatomical location of the TPO.R on T1W MRI. (C) *T*‐test of the ΔCBF value of TPO.R in the AD and MCI groups. (D) Anatomical location of the STG.R and MTG.R on T1W MRI. (E) *T*‐test of the ΔCBF of the STG.R and MTG.R in AD and MCI patients. L, left; MTG.R, right middle temporal gyrus; R, right; STG.R, right superior temporal gyrus; TPO.R, right temporal pole. ***: *p* < 0.001; **: *p* < 0.05.

**FIGURE 4:**
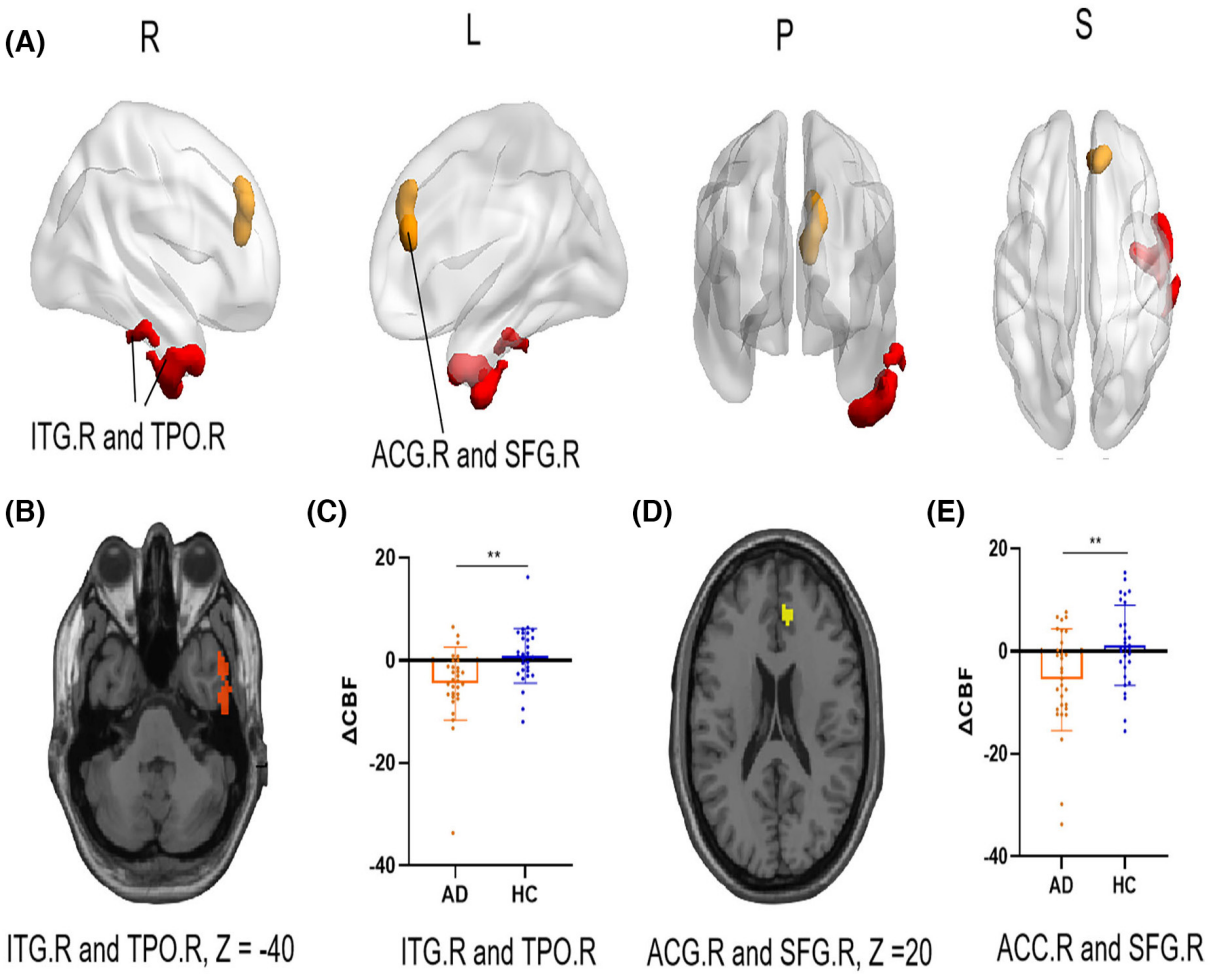
Significantly different ΔCBF areas between AD patients and HCs. (A) Surface‐based brain map of different ΔCBF areas between AD patients and HCs. (B) Anatomical location of the ITG.R and TPO.R on T1W MRI. (C) *T*‐test of the ΔCBF of the ITG.R and TPO.R in AD patients and HCs. (D) Anatomical location of the ACG.R and SFG.R on T1W MRI. (E) *T*‐test of the ΔCBF of the ACG.R and SFG.R in AD patients and HCs. ACG.R, anterior cingulate and paracingulate gyri; ITG.R, right inferior temporal gyrus; L, left; MTG.R, right middle temporal gyrus; R, right; SFG.R, Right superior frontal gyrus; STG.R, right superior temporal gyrus; TPO.R, right temporal pole; **: *p* < 0.05.

Compared with MCI patients, AD patients exhibited a stronger negative ΔCBF in the right anterior temporal lobe, including the right superior temporal gyrus and right middle temporal gyrus (Figure 3). Compared with HCs, AD patients had stronger negative ΔCBFs in the right anterior temporal lobe, including the right superior temporal gyrus, right middle temporal gyrus, and right inferior temporal gyrus (Figure 4). In addition, AD patients had stronger negative ΔCBF in the anterior cingulate and right superior frontal gyri than HCs. No significant difference in ΔCBF was observed between MCI patients and HCs.

### 3.4 Relationships between ΔCBF and cognitive function and vascular‐related factors

**FIGURE 5:**
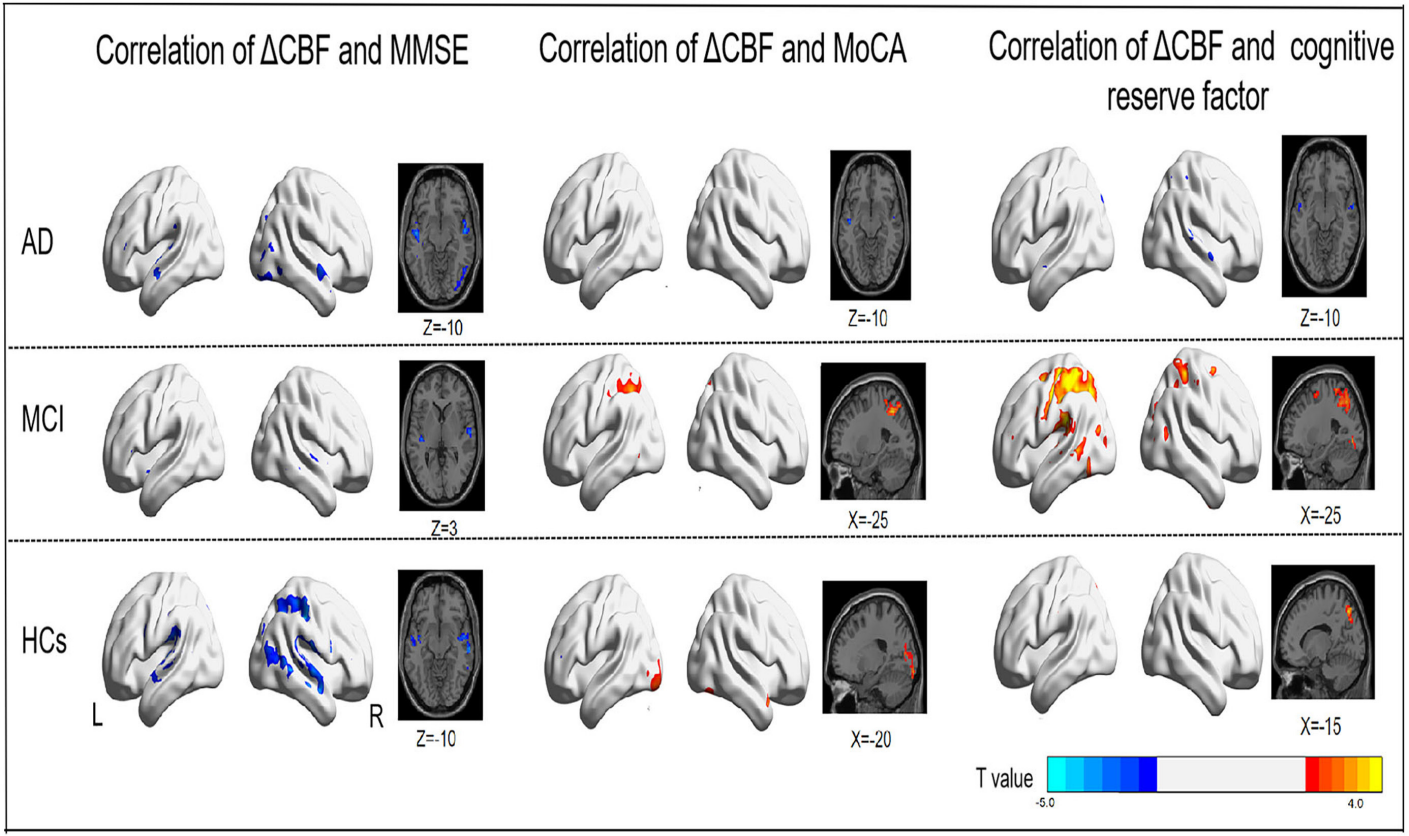
Correlation of the ΔCBF area with cognitive function, including MMSE, MoCA, and cognitive reserve factor scores, in AD patients, MCI patients, and HCs by voxelwise linear regression analysis after adjusting for age and sex (*T* value >2.3; *p* < 0.01, cluster size >30 voxels).

We performed voxelwise linear regression analysis on ΔCBF with the MMSE, MoCA, and cognitive reserve factors adjusted for age and sex (Figure 5 and Table S2). In AD patients, the ΔCBF in the bilateral anterior temporal lobe was negatively correlated with the MMSE score, MoCA score, and cognitive reserve factors. In correlation with the MMSE score, the ΔCBF in the bilateral anterior temporal lobe, including the bilateral superior temporal gyrus, middle temporal gyrus, and part of the occipital lobe, including the right inferior and middle occipital gyri, was negatively correlated with the MMSE score. The ΔCBF in the left superior temporal gyrus, which is part of the anterior temporal lobe, was negatively correlated with MoCA scores. The ΔCBFs in the right inferior temporal gyrus, right superior temporal gyrus, and left middle temporal gyrus were negatively correlated with cognitive reserve factors in AD patients.

In MCI patients, the ΔCBF in the bilateral superior temporal gyrus was negatively correlated with the MMSE score. The ΔCBF in the left parietal lobe, including the inferior parietal gyrus, superior parietal gyrus, and postcentral gyrus, was positively correlated with the MoCA score. The ΔCBF in the bilateral parietal lobe and occipital lobe was positively correlated with cognitive reserve factors. In HCs, the ΔCBF in the bilateral superior temporal gyrus and middle temporal gyrus was negatively correlated with the MMSE score. The ΔCBF in the bilateral part of the occipital lobe was positively correlated with the MoCA score, and the ΔCBF in the left superior parietal gyrus was positively correlated with cognitive reserve factors. There were no surviving clusters in the linear regression analysis when the FDR was corrected, and *p* < 0.05 and a cluster size >30 voxels were considered to indicate statistical significance.

Moreover, after adjusting for age and sex, we performed voxelwise linear regression analysis of ΔCBF and blood pressure, including the mean arterial pressure, systolic blood pressure, and diastolic blood pressure (Figure S1). Analysis of the correlation between the ΔCBF and mean arterial pressure revealed that the mean arterial pressure affected the ΔCBF in the anterior temporal lobe, including the superior temporal gyrus, in AD patients and HCs. In AD patients, the ΔCBF in the temporal lobe, including the left superior temporal gyrus, was positively related to the mean arterial pressure (Figure S1A). In HCs, the ΔCBF in the right superior temporal gyrus and left middle temporal gyrus was positively related to the mean arterial pressure (Figure S1A). In MCI patients, the ΔCBF in the bilateral occipital lobe, including the inferior and superior occipital gyri, was negatively correlated with the mean arterial pressure (Figure S1A). Analysis of the correlation between ΔCBF and systolic blood pressure (Figure S1B) or diastolic blood pressure (Figure S1C) revealed a similar correlation between ΔCBF and mean arterial pressure.

Finally, we explored the correlation between ΔCBF and vascular‐related factors, including BMI, the insulin resistance index (HOMA‐IR), fasting glucose, and C‐peptide, after adjusting for age and sex in AD patients, MCI patients, and HCs (Figure S2 and Table S3). A relationship between ΔCBF and vascular‐related factors was observed in AD patients but not in MCI patients or HCs. In AD patients, the ΔCBF in the right middle occipital gyrus and left middle frontal gyrus was positively correlated with BMI, and the ΔCBF in the left cerebellar anterior lobe was negatively correlated with insulin resistance. Moreover, the ΔCBF in part of the bilateral occipital lobe was negatively correlated with glucose, and the ΔCBF in the right superior temporal gyrus was positively correlated with C‐peptide. There were no correlations with the ΔCBF area after correction for BMI, fasting glucose, HOMA‐IR, or C‐peptide in the MCI patients and HCs.

## 4 DISCUSSION

The main finding of this exploratory experiment was that AD patients had a poor ability to maintain cerebral perfusion homeostasis under FMS stimulation. However, HCs were able to maintain cerebral perfusion homeostasis under FMS stimulation. Our study provides the first direct support for the concept that, compared with healthy older adults, patients with AD have different cerebral perfusion patterns in response to FMS according to neuroimaging. These findings provide a basic understanding of cerebral perfusion reactivity to FMS in AD patients. Moreover, compared with that of HCs, the anterior temporal lobe of AD patients is a distinct area that responds to FMS, and the ΔCBF in the anterior temporal lobe is correlated with cognitive function. ΔCBF in the anterior temporal lobe can be a potential radiological marker that reflects cognitive function in FMS patients. Collectively, these results will promote the development and implementation of interventions for AD via intermittent metabolic switching.

Negative ΔCBF in the extensive occipital cortex, parietal cortex, and part of the temporal lobar cortex in response to FMS was found in patients with AD, but ΔCBF remained statistically significant in no cerebral region in HCs. MCI patients had an intermediate ΔCBF change pattern in response to FMS between AD patients and HCs. Additionally, between‐group analysis revealed that AD patients had stronger negative ΔCBF in the right anterior temporal lobe than HCs. These results imply that AD patients are sensitive to FMS stimulation and have a poor ability to maintain cerebral perfusion homeostasis under stimulation. Additionally, HCs may have perfusion reserves to resist fluctuations and maintain homeostasis.

The possible mechanisms include the following factors. First, FMS induces neuronal adaptive responses, including metabolic state switching, and cellular molecular pathways that decrease CBF.^2^, ^6^, ^7^, ^8^ During FMS, neurons adapt from ketone‐dominant metabolism or low glucose metabolism to a glucose‐dominant metabolic pattern.^6^, ^7^ Elevated ketone body levels increase brain perfusion,^6^ and hyperglycemia decreases brain perfusion.^9^ Second, AD patients have disrupted energy homeostasis,^30^ as supported by the lower resting brain perfusion and glucose metabolic rates in AD patients than in HCs.^19^, ^31^, ^32^ This reduction may lead to hypoxia and a reduced ability to maintain metabolic homeostasis.^33^ Because of high brain energy metabolic deficits and reduced blood flow reserves in AD patients,^30^ FMS‐induced decreases in CBF lead to more robust effects in AD patients than in HCs. Third, an analysis of the relationships between ΔCBF and vascular‐related factors, including blood pressure, BMI, insulin resistance, fasting glucose, and C‐peptide, also indicated that ΔCBF may be more susceptible to vascular‐related factors in AD patients than in MCI patients or HCs. These findings suggest that the ΔCBF in AD patients is more susceptible to these vascular‐related factors. In contrast, the ΔCBF in HCs remains at homeostasis regardless of whether vascular‐related factors are elevated or decreased.

Compared with that in HCs, the anterior temporal lobe is a distinct area that responds to FMS in AD patients, and the ΔCBF in the anterior temporal lobe is correlated with cognitive function. ΔCBF in the anterior temporal lobe was negatively correlated with all three cognitive functions, MMSE, MoCA, and cognitive reserve factors, in AD patients and negatively correlated with MMSE scores in MCI patients and HCs. This finding was supported by previous studies showing that the anterior temporal lobe is an anatomical structure involved in AD. The volume of the anterior temporal lobe is positively correlated with cognitive function and decreases the functional connectivity and volume of the anterior temporal lobe involved in the progression of AD.^34^, ^35^

This was an exploratory study of the cerebral perfusion pattern response to FMS in AD patients using a relatively larger sample size of 92 participants (30 AD patients, 32 MCI patients, and 30 HCs) than that in previous studies of similar designs in young participants (with sample sizes ranging from 11 to 44).^20^, ^21^, ^22^, ^36^ A larger sample size is needed to confirm these findings in the future. However, this study has several limitations. First, this study was designed as a self‐comparison study to evaluate cerebral perfusion in fasting and postprandial states to reflect the cerebral response to FMS. Although previous studies have demonstrated high reliability and reproducibility for the same scanner,^37^ we cannot exclude the possibility that the results were at least partly attributable to the interaction between scan orders. In future studies, designing blank controls in which participants do not eat breakfast between the two CBF scans would help eliminate random fluctuations in CBF between fasting CBF and CBF 30 min after eating. Second, we tested the short‐term cerebral perfusion pattern response to FMS. The long‐term cerebral perfusion pattern effect and cognitive effect of FMS in individuals with AD require further study. Third, we did not evaluate the cervical or intracranial artery stenosis which is a factor that induce decreased lateral CBF. We have collected cerebrovascular risk factors including BMI, fasting glucose, insulin, HOMA‐IR, C‐peptide, hypertension, diabetes, hypercholesterolemia, and smoking that linked with vascular diseases. No differences on these risk factors were observed between groups, indicating little bias of current findings on CBF that are associated with vascular diseases. Therefore, we speculated that the cervical or intracranial artery stenosis has relatively small impact on the study results. However, in the future, the cervical or intracranial artery stenosis should be evaluated to accurate estimate the impact on the CBF.

## 5 CONCLUSIONS

This study provides novel evidence that AD patients have a poor ability to maintain cerebral perfusion homeostasis under FMS stimulation. The anterior temporal lobe is a distinct area that responds to fasting–postprandial metabolic switching in individuals with AD, and the ΔCBF in the anterior temporal lobe is negatively correlated with cognitive function.

## FUNDING INFORMATION

This research was funded by the National Key Research and Development Program of China (grant numbers 2021YFC2500100, 2021YFC2500103, and 2022YFC2009904), the National Natural Science Foundation of China (grant numbers 82071187 and 81870821), the Shanxi Province Science Foundation for Youths (202203021222360), and Guangzhou Planned Project of Science and Technology (202201020343). Open Project of Shanxi Key Laboratory of Brain Disease Control BDC202204.

## CONFLICT OF INTEREST STATEMENT

The authors declare no conflict of interest.

## Supporting information


Data S1.

## Data Availability

The data will be made available from the authors upon reasonable request.
