# Supplementary material for: Neuroimaging analysis reveals distinct cerebral perfusion responses to fasting‐postprandial metabolic switching in Alzheimer's disease patients
Source: CNS Neurosci Ther. 2024 Sep 11;30(9):e70014. doi: 10.1111/cns.70014 (PMC11388574; doi:10.1111/cns.70014)
Supplement: Supplementary file 1 — Data S1. [file CNS-30-e70014-s001.docx]

Supplementary material

**Table S1 The location of significant ΔCBF areas responding to fasting-postprandial metabolic switching in AD and MCI**

|  | | **AD** | | **MCI** | | | | |
| --- | --- | --- | --- | --- | --- | --- | --- | --- |
| **Brain lobe** | **AAL**  **atlas**  **index** | | **Brain regions** | **No. of**  **Voxels** | **Brain lobe** | **AAL atlas**  **index** | **Brain regions** | **No. of**  **Voxels** |
| Temporal Lobe | 81 | | Left superior temporal gyrus | 112 | Temporal Lobe | 82 | Right superior temporal gyrus | 12 |
|  | 82 | | Right superior temporal gyrus | 95 |  | 90 | Right inferior temporal gyrus | 42 |
|  | 84 | | Right temporal pole: superior temporal gyrus | 13 | Occipital lobe | 44 | Right calcarine fissure and surrounding cortex | 31 |
|  | 85 | | Left middle temporal gyrus | 114 |  | 46 | Right cuneus | 38 |
|  | 86 | | Right middle temporal gyrus | 145 |  | 47 | Left lingual gyrus | 20 |
|  | 88 | | Right temporal pole: middle temporal gyrus | 14 |  | 48 | Right Lingual gyrus | 13 |
|  | 89 | | Left inferior temporal gyrus | 36 |  | 49 | Left superior occipital gyrus | 88 |
|  | 90 | | Right inferior temporal gyrus | 28 |  | 50 | Rgiht superior occipital gyrus | 147 |
| Occipital lobe | 44 | | Right calcarine fissure and surrounding cortex | 23 |  | 51 | Left middle occipital gyrus | 215 |
|  | 46 | | Right Cuneus | 24 |  | 52 | Right middle occipital gyrus | 148 |
|  | 50 | | Right superior occipital gyrus | 134 |  | 53 | Left inferior occipital gyrus | 98 |
|  | 51 | | Left middle occipital gyrus | 254 |  | 54 | Right inferior occipital gyrus | 113 |
|  | 52 | | Right middle occipital gyrus | 264 |  | 56 | Right fusiform gyrus | 11 |
|  | 53 | | Left inferior occipital gyrus | 73 | Parietal lobe | 59 | Left superior parietal gyrus | 124 |
|  | 54 | | Right inferior occipital gyrus | 158 |  | 60 | Right superior parietal gyrus | 72 |
| Parietal lobe | 58 | | Right postcentral gyrus | 269 |  | 61 | Left iInferior parietal, but supramarginal and angular gyri | 30 |
|  | 59 | | Left superior parietal gyrus | 74 |  | 68 | Right precuneus | 38 |
|  | 60 | | Right superior parietal gyrus | 64 | Frontal lobe | 3 | Left superior frontal gyrus, dorsolateral | 14 |
|  | 62 | | Right inferior parietal, but supramarginal and angular gyri | 61 |  | 7 | Left middle frontal gyrus | 13 |
|  | 64 | | Right supramarginal gyrus | 44 | Cerebellum | 91 | Left cerebelum crus1 | 24 |
|  | 68 | | Right precuneus | 21 |  |  |  |  |
| Frontal lobe | 2 | | Right precentral gyrus | 48 |  |  |  |  |
|  | 7 | | Left middle frontal gyrus | 31 |  |  |  |  |
|  | 18 | | Right rolandic operculum | 18 |  |  |  |  |

Abbreviations: AAL, Anatomical Automatic Labeling.

**Table S2** **The location of ΔCBF area correlated with cognitive function among AD, MCI and HCs by linear regression analysis after adjusting age and sex.***

| **Group** | **Correlation** | **Cluster index** | | **Brain regions** | **No of voxels** |
| --- | --- | --- | --- | --- | --- |
| AD | ΔCBF and MMSE | 1 | Left parahippocampa gyrus | | 103 |
|  |  | 2 | Right inferior occipital gyrus | | 134 |
|  |  |  | Right middle occipital gyrus | |  |
|  |  | 3 | Right superior temporal gyrus | | 73 |
|  |  |  | Right middle temporal gyrus | |  |
|  |  | 4 | Left superior temporal gyrus | | 313 |
|  |  |  | Left middle temporal gyrus | |  |
|  | ΔCBF and MoCA | 1 | Left superior temporal gyrus | | 60 |
|  |  |  | Left middle temporal gyrus | |  |
|  | ΔCBF and cognitive reserve factor | 1 | Right inferior temporal gyrus | | 39 |
|  |  | 2 | Left middle temporal gyrus | | 45 |
|  |  | 3 | Right superior temporal gyrus | | 35 |
|  |  | 4 | Left superior occipital gyrus | | 35 |
| MCI | ΔCBF and MMSE | 1 | Left insula | | 45 |
|  |  |  | Left superior temporal gyrus | |  |
|  |  | 2 | Right superior temporal gyrus | | 58 |
|  |  | 3 | Right supramarginal gyrus | | 47 |
|  |  |  | Right postcentral gyrus | |  |
|  | ΔCBF and MoCA | 1 | Left inferior parietal gyrus | | 285 |
|  |  |  | Left superior parietal gyrus | |  |
|  |  |  | Left postcentral gyrus | |  |
|  | ΔCBF and cognitive reserve factor | 1 | Right lingual gyrus | | 88 |
|  |  |  | Right calcarine fissure and surrounding cortex | |  |
|  |  | 2 | Left inferior accipital gyrus | | 224 |
|  |  |  | Left fusiform gyrus | |  |
|  |  | 3 | Left postcentral gyrus | | 2182 |
|  |  |  | Left inferior parietal gyrus | |  |
|  |  | 4 | Right middle temporal gyrus | | 38 |
|  |  | 5 | Left middle frontal gyrus | | 38 |
|  |  | 6 | Right supplementary motor area | | 33 |
| HCs | ΔCBF and MMSE | 1 | Right superior temporal gyrus | | 1139 |
|  |  |  | Right middle temporal gyrus | |  |
|  |  |  | Right inferior parietal gyrus | |  |
|  |  |  | Right postcentral gyrus | |  |
|  |  |  | Right supramarginal gyrus | |  |
|  |  | 2 | Left superior temporal gyrus | | 81 |
|  |  |  | Left middle temporal gyrus | |  |
|  |  |  | Left precentral gyrus | |  |
|  |  |  | Left rolandic operculum | |  |
|  |  | 3 | Left inferior parietal gyrus | |  |
|  |  |  | Left postcentral gyrus | |  |
|  |  | 4 | Left cuneus | | 31 |
|  |  | 5 | Left inferior frontal gyrus | | 40 |
|  |  |  | Left precentral gyrus | |  |
|  |  | 6 | Right precuneus | | 86 |
|  | ΔCBF and MoCA | 1 | Right inferior occipital grus | | 220 |
|  |  | 2 | Left middle occipital grus | | 264 |
|  |  |  | Left inferior occipital grus | |  |
|  |  | 3 | Right calcarine | | 133 |
|  |  |  | Right cuneus | |  |
|  | ΔCBF and cognitive reserve factor | 1 | Left superior parietal gyrus | | 52 |

*Adjusted age and sex. Abbreviations: BMI: Body mass index; AD, Alzheimer’s disease; MCI, Mild cognitive impairment; HCs, Healthy controls; MMSE, Minimum Mental State Examination; MoCA, Montreal cognitive assessment; CBF, Cerebral blood flow; MNI, Montreal Neurological Institute.

**Table S3** **The location of ΔCBF area correlated with vascular related factors among AD, MCI and HCs by linear regression analysis after adjusting age and sex.**

|  | Cluster index | Brain regions | No of voxels | Peak T value | MNI Coordinates  X Y Z | | |
| --- | --- | --- | --- | --- | --- | --- | --- |
| ΔCBF of AD and BMI | 1 | Right middle occipital gyrus | 38 | 3.12 | 27 | -99 | 0 |
|  | 2 | Left middle frontal gyrus | 47 | 3.10 | -36 | 36 | 21 |
| ΔCBF of AD and insulin resistance | 1 | Left cerebellum anterior lobe | 34 | -3.22 | -78 |  |  |
| ΔCBF of AD and glucose | 1 | Right inferior temporal gyrus | 32 | 3.51 | 42 | -12 | -42 |
|  | 2 | Left inferior occioital gyrus | 60 | -3.51 | -42 | -63 | -12 |
|  | 3 | Right lingual gyrus | 49 | -2.93 | 21 | -87 | -9 |
|  | 4 | Left middle occioital gyrus | 76 | -3.13 | -33 | -75 | 15 |
|  | 5 | Left superior temporal gyrus | 34 | -3.20 | -45 | -39 | -6 |
|  | 6 | Left supraMarginal gyrus | 173 | -3.61 | -57 | -20 | 22 |
|  |  | Left postcentral gyrus |  |  |  |  |  |
| ΔCBF of AD and C-peptide | 1 | Right superior temporal gyrus | 34 | 2.93 | 42 | -21 | 6 |

Abbreviations: CBF: cerebral blood flow; ΔCBF: postprandial CBF subtracting fasting CBF; AD, Alzheimer’s disease; BMI: body mass index.

**
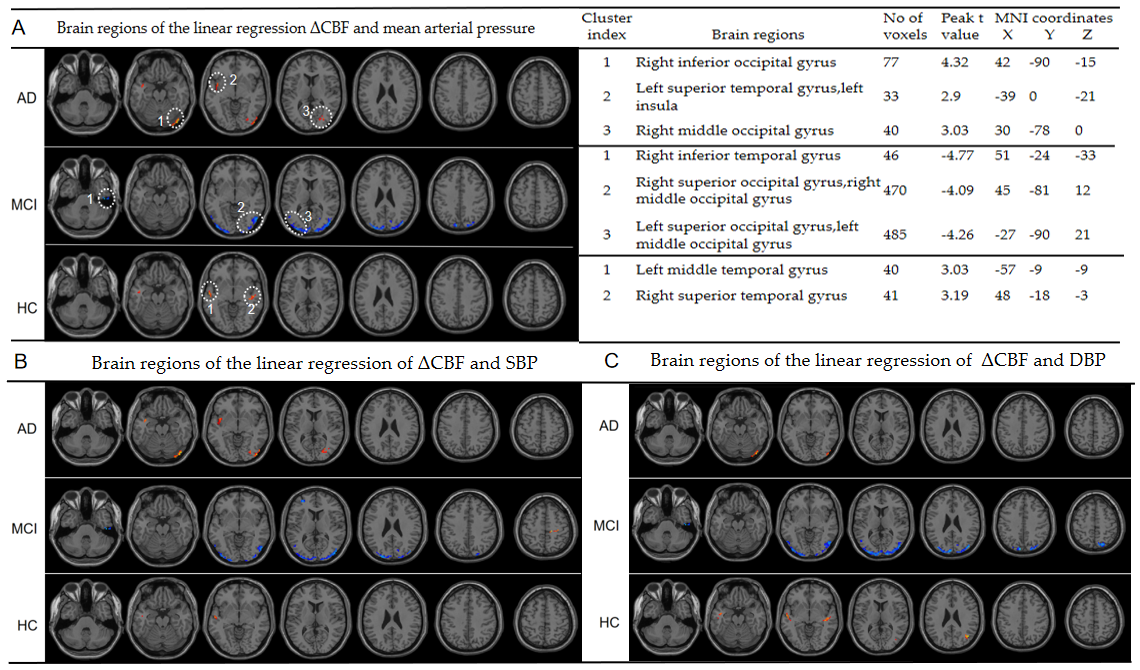
**Figure S1 The ΔCBF area correlated with blood pressure among AD, MCI, HCs by voxel-wise linear regression analysis adjusted age and sex (*P*<0.01, cluster >30 voxels). (A) The ΔCBF area correlated with and mean arterial pressure and location of the ΔCBF area. (B) The ΔCBF area correlated with and systolic arterial pressure. (C) The ΔCBF area correlated with and diastolic arterial pressure. Abbreviations: SBP, systolic blood pressure; DBP, diastolic blood pressure, MNI: Montreal Neurological Institute.


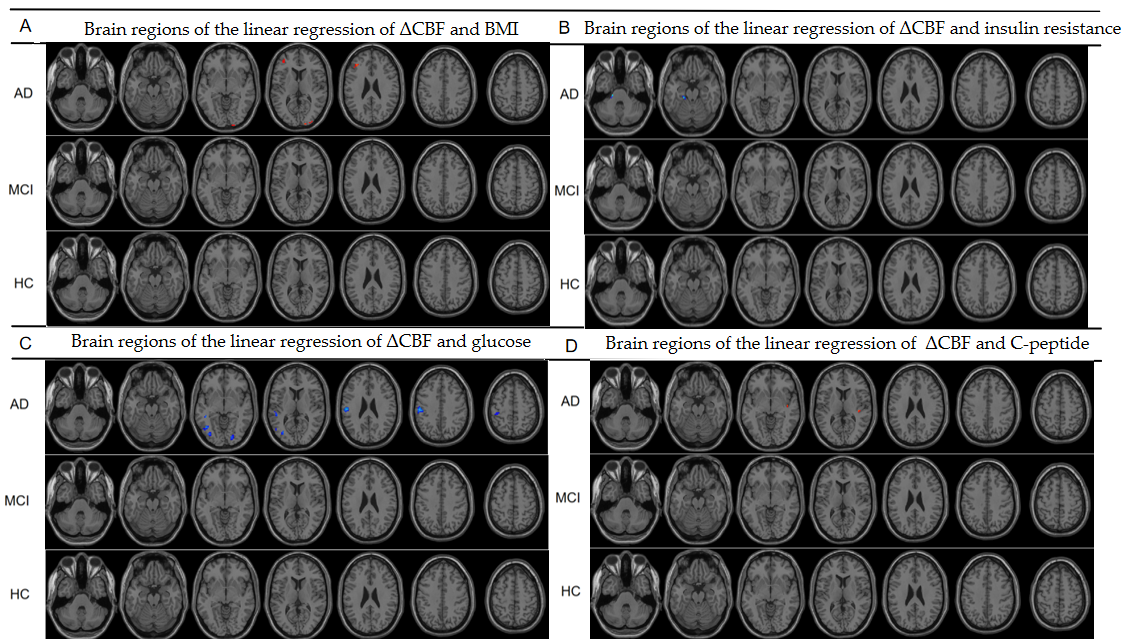


Figure S2 The brain regions of the linear regression of ΔCBF and vascular related factors among AD, MCI and HCs adjusted age and sex (*P*<0.01, cluster >30 voxels). (A) The brain regions of the linear regression of ΔCBF and BMI among AD, MCI participants and HCs. ΔCBF in right middle occipital gyrus and left middle frontal gyrus in AD had positive relationship with BMI. (B) The brain regions of the linear regression of ΔCBF and insulin resistance among AD, MCI participants and HCs. ΔCBF in left cerebellum anterior lobe showed negative relationship with insulin resistance in AD participants. (C) The brain regions of the linear regression of ΔCBF and glucose among AD, MCI participants and HCs. Bilateral occipital-dominant ΔCBF showed negative relationship with glucose in AD participants. (D) The brain regions of the linear regression of ΔCBF and C-peptide among AD, MCI participants and HCs. ΔCBF in right superior temporal gyrus showed positive relationship with C-peptide in AD participants. There was no significant region of ΔCBF with BMI, insulin resistance, glucose, C-peptide in MCI participants and HCs.

Note

The method of CBF to quantified to ml/100 g/min:

CBF images were produced using the default reconstruction pipeline (ReadyView) embedded in the GE-MR console (AW Server, GE). The pipeline performs the CBF calculation according to simple model with an assumption that the PLD is equal to ATT (REF Alsop 2015) and translates to the following equation by GE ReadyView’s user manual:

$$CBF=6000\times\frac{\lambda{\left( 1-e^{{-ST}/{T_{1t}}} \right)e}^{{PLD}/{T_{1b}}}}{2T_{1b}\left( 1-e^{{-LT}/{T_{1b}}} \right)\varepsilon\times{NEX}_{PW}}\times\frac{PW}{{SF}_{PW}PD}$$

Where:

$PW$ = perfusion weighted image from GE 3D ASL sequence

$PD$ = proton density image from GE 3D ASL sequence

$PLD$ = 2.025 seconds, post-labeling delay time chosen in this study

$LT$ = 1.45 seconds, labeling time

$\lambda$ = 0.9 mL/g, blood brain partition coefficent

$\varepsilon$ = 0.6, combination of labeling efficiency and background suppression pulse

$T_{1b}$ = 1.6 seconds, T1 relaxation time of blood at 3T

$ST$ = 2.0 seconds, saturation time

$T_{1t}$ = 1.2 seconds, T1 relaxation time of gray matter correction for proton density image

${NEX}_{PW}$ = 3, number of excitations (averaging) for perfusion weighted image

${SF}_{PW}$ = 32, signal factoring for perfusion weighted image
